# Population structure of a microparasite infecting *Daphnia*: spatio-temporal dynamics

**DOI:** 10.1186/s12862-014-0247-3

**Published:** 2014-12-04

**Authors:** Justyna Wolinska, Adam Petrusek, Mingbo Yin, Henrike Koerner, Jaromir Seda, Sabine Giessler

**Affiliations:** Leibniz-Institute of Freshwater Ecology and Inland Fisheries, Mueggelseedamm 301, 12587 Berlin, Germany; Department of Ecology, Faculty of Science, Charles University in Prague, Prague, Czech Republic; The Institute of Biodiversity Science, School of Life Science, Fudan University, Shanghai, 200433 China; Department of Neuroimmunology, Institut für Multiple Sklerose Forschung, Georg-August Universität, Göttingen, Germany; Biological Centre AS CR, Institute of Hydrobiology, Ceske Budejovice, Czech Republic; Department of Biology II, Ludwig-Maximilians-University Munich, Grosshaderner Str. 2, 82152 Planegg-Martinsried, Germany

**Keywords:** *Caullerya mesnili*, Host − parasite coevolution, ITS region

## Abstract

**Background:**

Detailed knowledge of spatial and temporal variation in the genetic population structure of hosts and parasites is required for understanding of host − parasite coevolution. As hot-spots of contemporary coevolution in natural systems are difficult to detect and long-term studies are restricted to few systems, additional population genetic data from various host − parasite systems may provide important insights into the topic. This is particularly true for parasites, as these players have been under-investigated so far due to the lower availability of suitable molecular markers. Here, we traced genetic variation (based on sequence variants in the internal transcribed spacer region, ITS) among seven geographically isolated populations of the ichthyosporean *Caullerya mesnili*, a common microparasite of the cladoceran *Daphnia* (here, the *D. longispina* hybrid complex). At three sites, we also studied parasite genetic variation over time (three to four sampling points) and tested for associations between parasite genotypes and host species.

**Results:**

Parasite (and host) populations were significantly structured across space, indicating limited dispersal. Moreover, the frequency of parasite genotypes varied significantly over time, suggesting rapid evolutionary change in *Caullerya*. However, the distribution of parasite genotypes was similar across different host species, which might in turn have important consequences for parasite epidemiology.

**Conclusions:**

The approach proposed here can be applied to track spatial and temporal changes in the population structure of other microparasite species for which sequence variation in the ITS or other highly variable genome regions has been documented but other types of polymorphic markers are lacking. Screening of parasite sequence variants allows for reliable detection of cross-species infections and, using advanced sequencing techniques in the near future, for detailed studies of parasite evolution in natural host − parasite systems.

**Electronic supplementary material:**

The online version of this article (doi:10.1186/s12862-014-0247-3) contains supplementary material, which is available to authorized users.

## Background

Host − parasite coevolutionary dynamics can be studied using experimental frameworks (e.g. [1-3]). However, given the increasing evidence that coevolution is highly sensitive to environmental variation, and thus possibly affected by experimental conditions (reviewed in [4,5]), it is crucial to assess the strength of coevolutionary interactions in the wild. Obtaining field data for coevolutionary dynamics presents practical difficulties, such as the necessity of long-term surveys. Despite that, field studies over time have been conducted within some natural host − parasite systems, e.g., in *Daphnia*–microparasites (e.g. [6-8]), chytrid–diatoms [9], bryozoans–myxozoans [10], plants–fungi (e.g. [11,12]), and freshwater snails–trematodes (e.g. [13,14]). In these and in many other field surveys to date, genetic variation over time has been investigated for the host alone, in most cases because of difficulties in obtaining suitable molecular markers for parasites. Given that time-lagged coevolution requires changes in the population genetic structure of both players [15,16], there is a need for studies directly tracking such changes in natural populations of parasites, in addition to their hosts.

Genetic studies of parasite populations over time have focused mainly on human parasites or economically important plant pathogens (e.g. [17-20]). For example, in fungal rust epidemics of cultivated flax, changes in allele frequencies were tracked at pathogen infectivity loci [21]. This deep insight at the allelic level became possible only because the genetics behind the plant − pathogen interactions had been studied for a long time (reviewed in [22,23]). Beyond the scope of such societally important pathogens, there has been relatively little work on genetic changes in parasite populations over time. Interactive effects among gene flow, local adaptation and the level of sexual reproduction will shape spatial and temporal population structures of parasites during host − parasite coevolution [24-26]. However, relevant studies in natural animal − microparasite systems have become feasible only recently, due to progress in the development of molecular markers for parasites.

One of the recently established models for host − parasite coevolution involves waterfleas of the genus *Daphnia* (Crustacea: Cladocera), key components of zooplankton in many lakes and ponds across the globe, and their microparasites (e.g. [8,27]). In natural populations of *Daphnia*, however, temporal changes within gene pools, resulting from host − parasite coevolution, have thus far been studied for host populations only (e.g. [6,28]). For example, it has been observed that the most common *Daphnia* genotypes decreased in frequency over successive generations across several heavily infected host populations, whereas such decreases were not detected in uninfected populations [7]. These results are consistent with the idea that parasites track common host genotypes and, consequently, exert negative frequency-dependent selection on their hosts [13,16]. We do not know, however, if and how the apparent changes in the relative genotype frequencies of hosts trigger responses in the relative genotype frequencies of parasites infecting these populations. Although genetic markers, such as allozymes (e.g. [6,7,28]) or, more recently, microsatellites (e.g. [29,30]), have been widely applied to track changes in genotype frequencies in natural *Daphnia* populations, markers for their microparasites are in a developmental phase [31,32].

In the present study, we analysed infections of *Daphnia* species from the *D. longispina* hybrid complex. In Europe this complex includes, among other taxa, the widespread and ecologically important species *D. cucullata*, *D. galeata* and *D. longispina*, as well as their interspecific hybrids [33,34]. These *Daphnia* are frequently infected by a variety of parasites (e.g. [35]). Among these, the protozoan *Caullerya mesnili* (class Ichthyosporea, order Ichthyophonida, [36]) is particularly common. *Caullerya* forms spore clusters in the gut epithelium of *Daphnia* [36]. This parasite is likely to cause significant selection pressure on *Daphnia* populations because of its high virulence (up to 95% fecundity reduction, [36,37]). Indeed, our previous laboratory and field results indicate that *Caullerya* can alter host genetic structures, both at the community and population levels [29,37]. For example, in artificial communities consisting of a mixture of several genotypes from two *Daphnia* species, the competition outcome between species and genotypes differed between *Caullerya*-infected and non-infected treatments [37]. Since *Caullerya* causes apparent changes in its host genetic structure, this parasite might, in turn, respond genetically to its changing host. To address potential genetic responses in populations of *Caullerya,* polymorphism in the internal transcribed spacer region (ITS1) of ribosomal DNA can be used [36,38]. Specifically, applying this ITS marker revealed that *Caullerya* populations collected from three geographically isolated lakes were distinct from each other [38].

Here, we applied the same ITS marker to study genetic changes in *Caullerya* populations in more detail. We studied parasite genetic variation over space (seven geographically isolated sites). We expected to observe substantial spatial isolation among parasite populations, as *Caullerya* appears to have no transmission vector other than its passively dispersing *Daphnia* hosts [39]. We then followed changes in parasite population structure over time (five of the seven sites were sampled over a period of two to five years). We hypothesized that *Caullerya* populations should change genetically, to remain infective to their ever-changing host. For *Caullerya* populations from three of the studied sites, we simultaneously collected genetic data from their individual *Daphnia* hosts. At this point, we aimed to compare the distribution of parasite genotypes across different coexisting host *Daphnia* species and their hybrids. We expected that host species would exhibit differing spectra of parasite genotypes, as previous experimental studies showed a varying likelihood of infection when these species were exposed to *Caullerya* [37].

## Results

### Parasite: spatio-temporal variation

**Figure 1:**
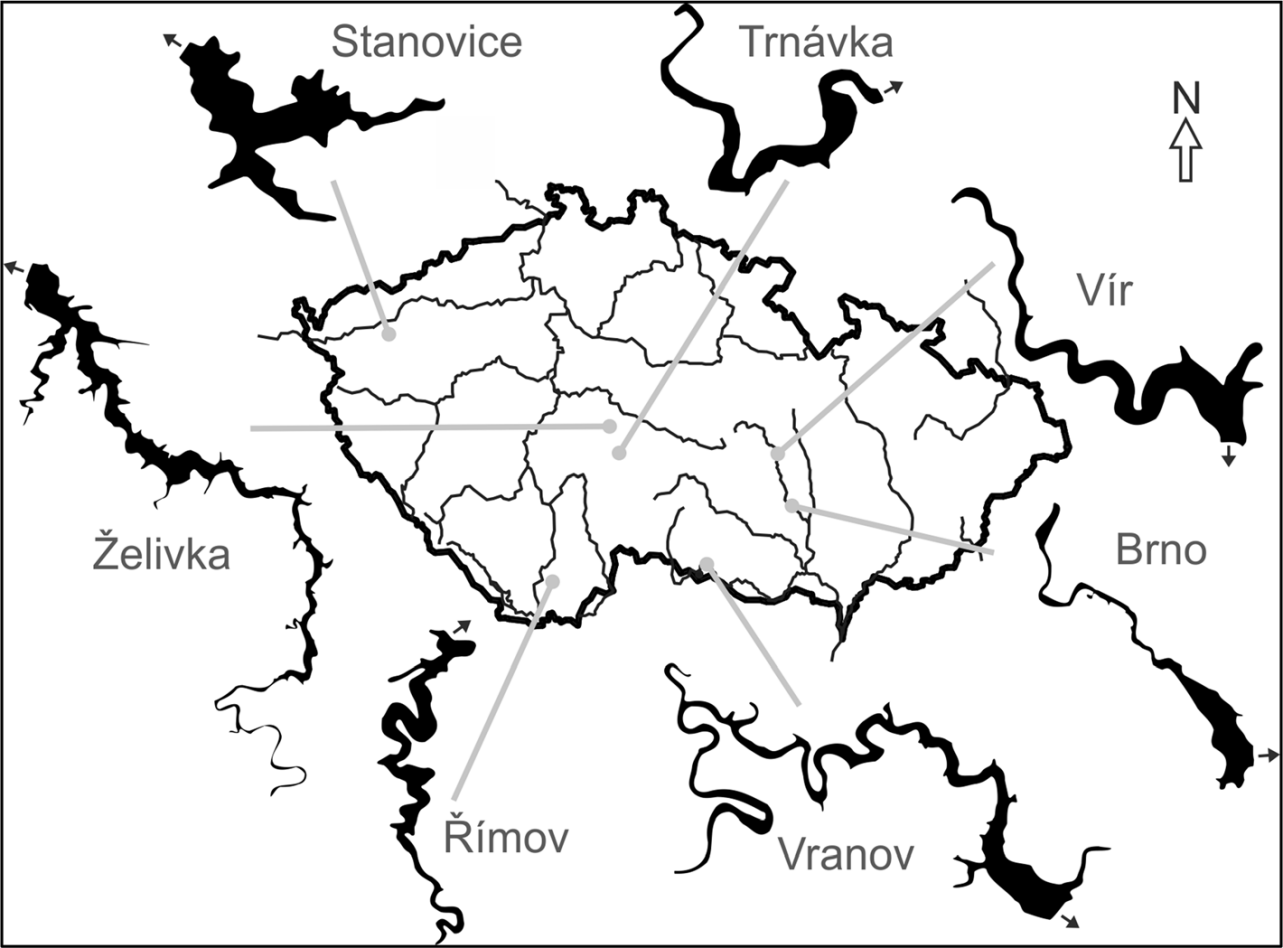
**Location of sampling sites in the Czech Republic and schematic outlines of their morphology.** A small arrow indicates the position of the dam and outflow of each reservoir. Map modified after Seda et al. [57].

**Figure 2:**
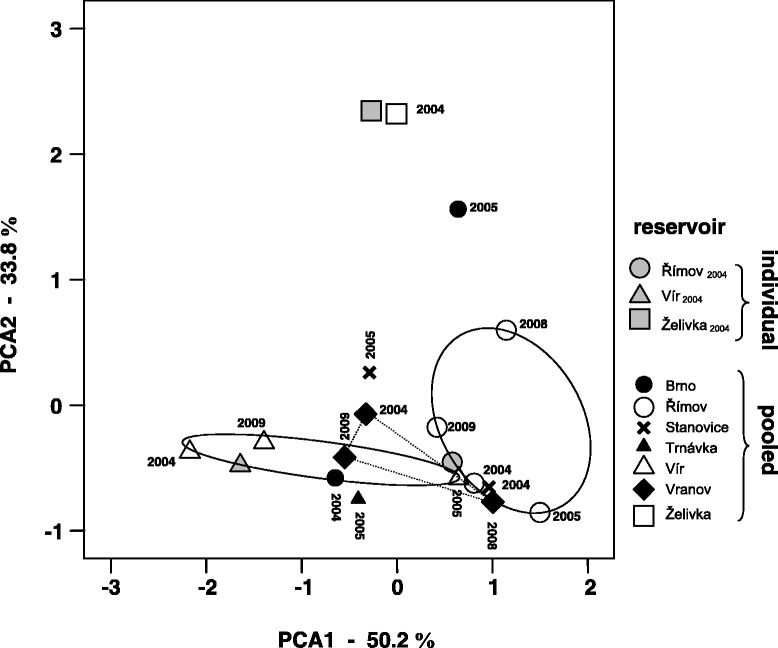
**Spatio-temporal population structure of** ***Caullerya mesnili*** **parasites.** Principal component loadings on the PCA-axes are based on the frequency of parasite TCS-types; the first two axes account for 84% of the variation in the data. Parasite DNA was obtained from *Daphnia* hosts originating from seven lakes; some of the populations were sampled over a period of two to five years, resulting in 16 samples altogether (genotyped after pooling of 20 infected host, per sample; see Table 1). If the same lake was sampled 3–4 times, the samples are connected by a line. Three additional subsamples, for which 10 infected individuals (per population) were genotyped individually (Table 1), are shown in grey.

Altogether, 16 zooplankton samples, collected across seven water reservoirs in the Czech Republic (see Figure 1), were used for *Caullerya* genotyping. All 16 samples had been genotyped after pooling of 20 infected hosts, per sample. Additionally, three of those samples were genotyped on the individual level: ten infected hosts per sample [40]. In total, 216 unique *Caullerya* ITS sequence variants were identified among 795 obtained ITS sequences. These ITS variants were further assigned by statistical parsimony network analysis to ten different representative sequence variants, TCS-types (C1 to C10 Table 1; for more details see [40]); 40% of sequences were classified to the most abundant TCS-type, C6. In contrast, four TCS-types (C3, C7, C9 and C10) were present just once among 795 analysed sequences [40] and were therefore excluded from subsequent analyses. The spatial and temporal population structure of parasites is displayed in a PCA plot based on frequencies of specific TCS-types in 19 parasite population samples (Figure 2). The Římov, Vír and Želivka samples from 2004, which were analysed from pooled host DNA, clustered together with the subsamples from the same three populations processed independently by analysing parasite DNA from single hosts, confirming that both methodological approaches result in comparable patterns see [40].

**Table 1:** **Summary of analysed** ***Caullerya mesnili*** **samples from seven lakes in the Czech Republic**

**DNA**	**Lake**	**Year**	**TCS-type**										**No. of ITS**
**Extraction**			**C1**	**C2**	**C3**	**C4**	**C5**	**C6**	**C7**	**C8**	**C9**	**C10**	**Sequences**
**20 pooled hosts/lake sample** ^**a**^													
	Brno	2004	6	0	0	10	0	11	0	0	0	0	27
	Brno	2005	2	0	0	12	1	2	0	14	0	1	32
	Římov^c^	2004	3	0	0	19	0	7	0	1	0	0	30
	Římov	2005	2	0	0	22	0	4	0	0	1	0	29
	Římov	2008	0	0	0	15	0	4	0	10	0	0	29
	Římov	2009	3	0	0	15	0	8	0	4	0	0	30
	Stanovice	2004	3	0	0	18	0	5	0	1	0	0	27
	Stanovice	2005	3	0	0	11	1	10	0	0	0	0	25
	Trnávka	2005	8	0	0	12	0	7	0	0	0	0	27
	Vír^c^	2004	9	0	0	2	0	17	0	0	0	0	28
	Vír	2005	3	0	0	15	0	6	0	1	0	0	25
	Vír	2009	4	0	0	5	0	17	0	0	0	0	26
	Vranov	2004	2	0	0	10	0	14	0	3	0	0	29
	Vranov	2008	3	0	0	22	0	7	0	0	0	0	32
	Vranov	2009	3	0	0	12	0	18	0	0	0	0	33
	Želivka^c^	2004	1	0	0	8	2	6	0	11	0	0	28
		∑	55	0	0	208	4	143	0	45	1	1	457
**10 single hosts/lake sample** ^**b**^													
	Římov^c^	2004	19	3	0	66	0	20	0	9	0	0	117
	Vír^c^	2004	34	1	1	20	0	56	0	0	0	0	112
	Želivka^c^	2004	10	0	0	26	7	22	1	43	0	0	109
		∑	63	4	1	112	7	98	1	52	0	0	338

Specifically, the number of ITS sequences obtained from *C. mesnili* parasite DNA, as well as their assignment to representative sequence variants (TCS-types) by statistical parsimony analysis (C1-C10, the labels are consistent with [38]) are provided. Parasite DNA was either extracted from 10 individual *Daphnia* hosts per sample or 20 pooled *Daphnia* hosts per sample. Table modified after Giessler and Wolinska [40].

^a^Parasite data from Giessler and Wolinska [40].

^b^Parasite data from Wolinska et al. [38].

^c^Parasite DNA from these samples was analysed using both approaches (i.e. 10 single hosts and 20 pooled hosts were genotyped, respectively).

Parasite populations from different lakes were in most cases separated and the spatial separation usually persisted over time. However, populations from different lakes sometimes clustered together at the same point in time (e.g. Římov 2004 and Stanovice 2004) or after a shift in time (Římov 2004 and Vír 2005). The extent of the spatial and temporal separation of *Caullerya* populations was supported by hierarchical analysis of molecular variance (AMOVA tests). The spatial (i.e., among lakes) variance component was significant in all three cases analysed (years 2004, 2005 and 2009), explaining up to ~22% of the parasite genetic diversity (Table 2). The temporal (i.e., among years) component was also significant in all three cases (for Římov, Vír and Vranov), and explained up to 12% of the variation in the parasite data (Table 2).

**Table 2:** **Results of AMOVA to explore spatial and temporal population structure in** ***Caullerya mesnili*** **parasites**

**Lake samples**	**Source of variation**	**df**	**Percent variation**	***P*** **-value**	
**Spatial variation**					
All 2004	Among lakes	5	18.5	<0.001	***
	Within lake	163	81.5		
All 2005	Among lakes	4	21.9	<0.001	***
	Within lake	133	78.2		
All 2009	Among lakes	2	8.9	0.0029	*
	Within lake	86	91.1		
**Temporal variation**					
All Římov	Among years	3	9.4	0.001	**
	Within year	114	90.6		
All Vír	Among years	2	12.5	0.001	**
	Within year	76	87.5		
All Vranov	Among years	2	7.3	0.0039	*
	Within year	91	92.7		

Calculations were based on the frequency of representative ITS-sequence variants (TCS-types) in *C. mesnili* parasite DNA (20 pooled *Daphnia* hosts per sample; for selection of samples see Table 1 and main text). Significance levels from separate analyses to test for structures in space and time, respectively, were Bonferroni corrected (adjusted *P*-values: *P <0.05; **P <0.01; ***P <0.001).

### Parasite and host: genetic associations

**Figure 3:**
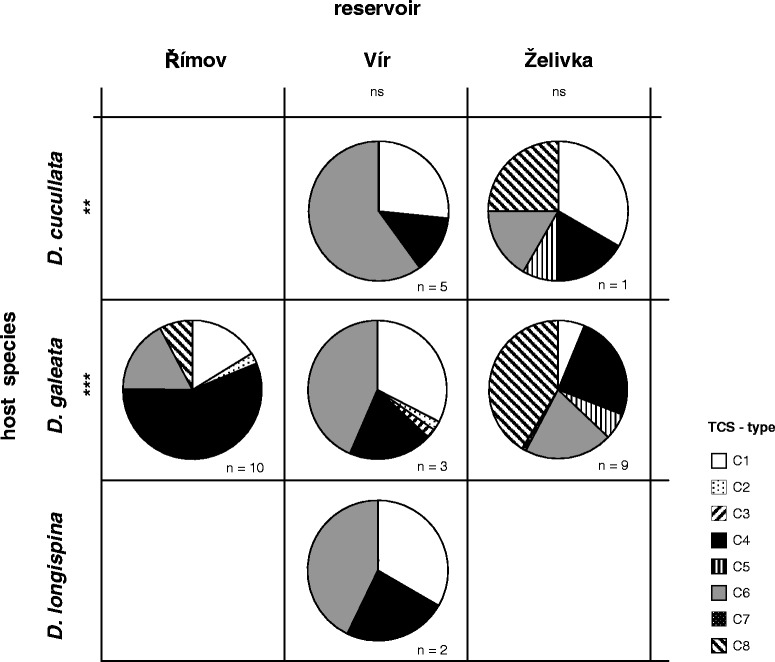
**Distribution of representative ITS-sequence variants (TCS-types) in** ***Caullerya mesnili*** **.** Parasite DNA was isolated from *Daphnia* hosts belonging to three different species (10 infected *Daphnia* per lake were genotyped individually; Table 1), and sampled across three different lakes. Species identity of *Daphnia* hosts was derived from the allelic variation at 15 microsatellite loci (see Additional file 1: Figure S1). Distribution of TCS-types varied among lakes but not among host species: n: number of host individuals per lake and species; **P <0.01, ***P <0.001 (Fisher’s exact tests).

Parasite ITS-data were obtained from individual *Daphnia* hosts selected from Římov, Vír and Želivka, sampled in 2004. These individual *Daphnia* (i.e., 3 lakes × 10 individuals) were analysed at 15 microsatellite loci. The position of *Daphnia* individuals relative to reference genotypes in the FCA plot (Additional file 1: Figure S1) revealed that these individuals belonged to three species, *D. cucullata*, *D. galeata* and *D. longispina*, and no hybrids were detected in this small dataset. Overall, the distribution of parasite representative sequence variants (TCS-types) was more similar across different host species originating from the same lake than within host species from different lakes (Figure 3). This was confirmed by AMOVA, where the degree of variation explained by the differences among host species was negligible (<0.01%, Table 3), indicating that all host species were infected by the same array of parasite genotypes.

**Table 3:** **Results of AMOVA to explore the association between** ***Caullerya mesnili*** **parasite genotypes and the genetic (i.e., species) identity of** ***Daphnia*** **hosts**

**DNA extraction**	**Source of variation**	**df**	**Percent variation**	***P*** **-value**
**10 single hosts/lake sample**				
	Among lakes	2	25.2	0.013
	Among host species, within lake	3	−0.1	0.32
	Within host species	332	74.9	<0.001

*Daphnia* species identity was assigned using 15 microsatellite loci (see Additional file 1: Figure S1). Calculations were based on the frequency of representative ITS-sequence variants (TCS-types) in *C. mesnili* parasite DNA (10 individually genotyped *Daphnia* hosts per sample; for selection of samples see Table 1).

### Host: spatial variation

Out of the larger subset of randomly selected *Daphnia* from Římov, Vír and Želivka, sampled in 2004 (i.e., ~50 individuals per lake), the only host species present in all three lakes was *D. galeata* (47 individuals from Římov, 23 from Vír and 9 from Želivka). AMOVA tests revealed that a significant amount of genetic variation in the *D. galeata* dataset was explained by the among-lakes component (14%, see Table 4). Thus, *D. galeata* populations were significantly structured across space.

**Table 4:** **Results of AMOVA to explore the spatial population structure in host species (** ***Daphnia galeata*** **)**

**Source of variation**	**df**	**Percent variation**	***P*** **-value**
Among lakes	2	13.9	<0.001
Among individuals, within lake	155	86.1	

Calculations were based on the frequency of alleles at 10 microsatellite loci in *Daphnia* host DNA. DNA was extracted from *D. galeata* representing a random host population sample, i.e., consisting of uninfected and infected individuals originating from three lakes sampled in 2004 (Římov, Vír and Želivka; see Table 1).

## Discussion

The reservoirs studied are geographically isolated, preventing any direct dispersal of *Daphnia* hosts or their parasites through flowing water. Indeed, we show here that *Caullerya* populations originating from seven lakes were significantly structured across space, as were their host populations (at least *D. galeata*, a species for which sufficient data were available). The latter finding is consistent with previous studies on the *D. longispina* complex; *Daphnia* populations from different lakes are often genetically differentiated even within a single region at relatively small spatial scales (e.g. [41-43]). The major dispersal pathway for the host, *Daphnia*, is most likely via transport of their diapausing eggs by water birds, which are enclosed in a protective structure called an ephippium [44,45]. Immigrant genotypes, however, have a low likelihood of becoming established in already developed populations [41,46,47]. In the case of *Caullerya*, their spread seems not to be linked directly to this major dispersal stage of the host, diapausing eggs, because eggs and embryos of infected mothers are not themselves infected [39]. Instead, if infected *Daphnia* are ingested by waterfowl, it is likely that *Caullerya* spores (but not *Daphnia*) could survive the passage through the bird’s gut because of a thick and robust spore wall [36], and then be excreted into another lake. Alternatively, infective stages of parasites might be transported independently of the *Daphnia* host on the body surface of waterfowl. However, even if parasite spores are spread by birds in this way, such dispersal pathways would need to provide infective doses high enough for successful transmission (e.g. [48]).

The significant genetic differentiation of spatially separated *Caullerya* populations observed here might also be influenced by processes other than limited gene flow. In particular, temporal changes within *Caullerya* populations, as were observed in the studied reservoirs, may cause rapid genetic divergence. Moreover, environmental differences among the studied lakes such as the trophic level (see [35]) might promote different parasite genotypes (i.e., genotype-by-environment interactions, a phenomenon reported for a number of *Daphnia*-microparasite systems, reviewed in [4]). Finally, a low level of sexual reproduction (and recombination) in ichthyosporeans [49,50] might also result in a high level of population structuring.

In addition to significant spatial genetic variation, we found support for temporal variation in *Caullerya*. In lakes Římov and Vír, for example, the genetic composition of *Caullerya* populations varied substantially during the five-year study period. Again, it cannot be excluded that the observed temporal changes are not only a result of adaptation of the parasite to its changing host, but fluctuating environmental conditions might play a role too. Indeed, genotype-by-environment interactions have been previously observed in the *Daphnia* − *Caullerya* system [51]. As far as we know, studies looking at temporal changes in microparasite populations infecting wild animal host populations are still rare (but see [20]). Thus, the *Daphnia* − *Caullerya* system appears to be an attractive model for studying host − parasite coevolution in the wild, because of its accessibility (*Caullerya* infections are common across European *Daphnia* populations, [35,37]), the short generation time of both players and, finally, the insights already available from molecular markers.

Another important finding of our study is that different co-existing *Daphnia* host species were all infected by the same genotypic array of the parasite *Caullerya*. This is surprising at first sight, because a prerequisite for parasite-driven, negative frequency-dependent selection is a strong genetic specificity for infection, resulting in genetically different hosts being infected by genetically different parasite strains [52]. In other words, parasite populations should be well structured with respect to the host genotypes they can infect (e.g. [53]). Despite this, patterns similar to our results have already been observed elsewhere. For example, tight interactions between parasite and host genotypes were reported in a bumblebee–trypanosome system at the level of spatially isolated colonies within a single host species [54], but identical parasite clones were found across several coexisting bumblebee species [55]. Such horizontal transmission of parasites was argued to be favoured by niche overlap between bumblebee host species [56]. This might also be the case in the *D. longispina* system studied here, where different *Daphnia* species coexist despite some differences in their ecological preferences [57,58]. Another mechanism that might facilitate the homogenous distribution of *Caullerya* genotypes between different *Daphnia* species is host hybridization. Specifically, *Daphnia* hybrids might serve as ‘stepping stones’ that allow parasites to switch among host species (i.e., the ‘hybrid bridge’ model, [59]). Regardless of its cause, multi-host species infection might have important consequences for parasite epidemiology. Theory predicts that parasites which do not depend on the population dynamics of a single host species should consequently be able to maintain large effective population sizes and high evolutionary potential [60,61], a prediction further supported by empirical work [62].

Interestingly, previous field and experimental surveys have shown that coexisting *Daphnia* host species and even genotypes differ in their likelihood of becoming infected with *Caullerya* (e.g. [29,37]). Still, as indicated by present genetic results, same parasite genotypes seem to be able to establish across various *Daphnia* species. However, we should take the latter observation with caution, because the conclusions are based on screening of a limited number of samples and host individuals. Thus, this hypothesis should be further tested, for example by conducting genetic analyses of a high number of infected and randomly chosen *Daphnia* from lakes where different *Daphnia* species coexist which is a common phenomenon (e.g. [58]). If a sufficient number of suitable samples from *Caullerya*-infected populations is obtained, host species can be assessed for the likelihood of infection (by comparing a distribution of species in infected and random groups of hosts, see [37]), and parasite genotypic arrays among host species can be compared to test for potential specificity once the host defence is broken. Experimental tests should follow as well, where different *Daphnia* host species are exposed to a mixture of parasite genotypes, and the genotypic array of successful parasites is compared among these hosts (see [63]).

Finally, the molecular approach used in this study, involving the combination of a transformed ITS dataset and DNA-pooling of a moderate number of infected host specimens, proved to be a simple but reliable method to address parasite population differentiation over space and time (see also [40]). In the long term, however, results derived from a multi-copy gene should be confirmed by single-copy molecular markers (e.g. [64]). One also has to keep in mind that we have used rather neutral genetic markers, both for *Daphnia* (microsatellites) and for *Caullerya* (ITS region). Although these markers would still be linked to loci under selection in our (mainly) clonal host − parasite system, the outcome of coevolution actually depends on the loci coding for resistance in hosts and infectivity in parasites see (e.g., [21,65,66]). Nevertheless, in terms of the parasite *Caullerya*, ITS is the only available marker to date. The ITS dataset generated here, by standard Sanger sequencing, may in future serve as a useful reference for higher-resolution datasets obtained from more powerful markers and advanced sequencing technologies [67-69].

## Conclusions

In summary, by analysing ITS sequences, we successfully addressed spatial and temporal variation in the genetic structure of the ichthyosporean microparasite *Caullerya mesnili* infecting the cladoceran *Daphnia* host. Our results suggest that *Caullerya* can evolve rapidly and is thus likely to adapt to its hosts, as we detected significant genetic variation in parasite populations across space and time. When upcoming molecular and bioinformatic tools allow changes in genotype frequencies of parasites and hosts to be tracked together on a large scale and over the long term, the *Daphnia* − *Caullerya* system might become a useful model for host − parasite coevolution in the wild. Moreover, the methods proposed here allow access to molecular studies of other microparasite species, for which sequence variation in the ITS or other highly variable genome regions has been documented but other types of polymorphic markers are lacking.

## Methods

### Study sites

In a previous work, *Daphnia* communities from eleven drinking water reservoirs in the Czech Republic were screened for parasite prevalence in summer and autumn of 2004 and 2005, across three stations along each reservoir’s horizontal axis: upstream, middle and downstream [35]. The *Daphnia* hosts in these lakes are represented almost exclusively by members of the *D. longispina* complex [29,34]. The parasite *Caullerya mesnili* was abundant in seven of these lakes (Brno, Římov, Stanovice, Trnávka, Vír, Vranov and Želivka), mainly in autumn and at the upstream sites, infecting up to 40% of the entire *Daphnia* community [35]. The position of the seven lakes is indicated on a schematic map (Figure 1), basic limnological characteristics are provided in [57]. For the present study, we used ethanol-preserved samples of *Daphnia* populations already collected for other purposes. Collections were taken at all seven lakes in autumn of 2004 and 2005 [35], as well as at three of these lakes (Římov, Vír and Vranov) in autumn of 2008 and 2009.

### Sample selection

From all collected zooplankton samples, we selected those containing a substantial proportion of *Caullerya*-infected *Daphnia*, for analyzing parasite genetic variation. This resulted in 16 zooplankton samples across seven locations, 1 to 4 per lake (Table 1); mainly upstream samples were selected (with two exceptions: Brno 2005 – downstream sample, Stanovice 2005 – middle sample). It was impossible to obtain a more balanced sample set, as the prevalence of *Caullerya* varies unpredictably from year to year [35]. All 16 zooplankton samples served to analyze the spatio-temporal variation (here, parasite DNA was extracted from pooled-host, see below). Three of the 16 samples were additionally used to analyze the genetic association between parasites and hosts (parasite DNA was extracted from individual-host). Thus, 19 parasite population samples were obtained in total.

#### Parasite: spatio-temporal variation

We used *Caullerya* ITS data previously obtained from all 16 samples in a methodological study developing an efficient approach to analysis of ITS sequences [40]. From each of 16 samples, 20 *Caullerya*-infected *Daphnia* were pooled before DNA extraction.

#### Parasite and host: genetic associations

We used additional *Caullerya* ITS data obtained from Římov, Vír and Želivka, sampled in autumn 2004 [35]. DNA was extracted individually from 10 *Caullerya*-infected *Daphnia* hosts per lake (Table 1). Whereas only parasite genotypes were analysed previously [35], for the purpose of this study, we determined the corresponding host genotypes from the respective DNA samples (through analyses of 15 microsatellite loci; 3 lakes × 10 infected individuals).

## Host: spatial variation

We randomly selected ~50 additional *Daphnia* individuals (including both infected and non-infected individuals) from Římov, Vír and Želivka, sampled in autumn 2004, and determined host genotypes from the respective DNA samples (through analyses of 10 microsatellite loci).

### Genotyping

#### Parasite

Both ITS data sets (obtained by DNA extraction from pooled or single hosts, see Table 1) have recently been used for validation of DNA pooling and for comparison of different statistical methods identifying representative ITS-sequence variants (i.e., statistical parsimony networks vs. neighbour-joining analysis, [40]). Consequently, all molecular procedures concerning genomic DNA extraction, PCR conditions, cloning and Sanger sequencing are described in detail elsewhere [38,40]. In short, for 16 population samples genotyped after pooling of 20 infected host individuals, between 25 and 33 ITS sequences were obtained per population sample, while for the three population samples where parasites were analysed on the level of single hosts, between 109 and 117 ITS sequences were obtained per population sample (about 10 sequences per individual host; Table 1). Both sets of sequences were of ca. 600 bp in length. Sequences were aligned in BioEdit [70] using the ClustalW algorithm and the alignment was then corrected by hand when necessary. Then, in order to address relevant genetic polymorphism in the ITS multi-copy region, we assigned slightly different sequences to the representative variants, using statistical parsimony network analysis as implemented in TCS 1.21 [71]. Specifically, we applied a cut-off of three connection steps (gaps were considered as a fifth base; each 1 bp indel was scored). This approach is described in detail and justified in Giessler and Wolinska [40]. All further analyses of the parasite data were then based on the dataset including only representative ITS-sequence variants (“TCS-types”).

*Host. Daphnia* individuals were genotyped at polymorphic microsatellite loci [72]. First, to analyse the distribution of parasite genotypes across different host species or hybrids, the same genomic DNA that had been isolated from 30 infected *Daphnia* specimens (used for parasite genotyping on the level of single host individuals) was genotyped at 15 microsatellite loci. These hosts originated from Římov, Vír, and Želivka, sampled in 2004 (10 individuals per lake, Table 1). List of loci and molecular protocols are described elsewhere [43]. *Daphnia* individuals were then assigned to different host species or hybrids, by a factorial correspondence analysis (FCA) in GENETIX 4.05 [73]. As a reference, 49 well-defined genotypes (same as in [43]) were used, representing each of the three dominant parental species in the studied reservoirs (i.e., *D. cucullata*, *D. galeata* and *D. longispina*), as well as their interspecific hybrids. Thus, we obtained joint information on the taxonomic classification of 30 host individuals (microsatellite data) and the genotypes of parasites (ITS data) infecting each of these *Daphnia* hosts. Second, to study the extent of among-population genetic differentiation in hosts, we genotyped approximately 50 randomly selected *Daphnia* individuals from each of the three aforementioned lakes, sampled in 2004, at 10 of the above used 15 microsatellite loci (same set of microsatellites as in [29]). This random sample consisted of both uninfected and infected individuals, thus representing the whole host population. Since, in the random population sample, only two parental species were present but several individuals could not be assigned unambiguously to clusters defined by the reference clones (data not shown), taxon membership was further evaluated by a Bayesian method in NewHybrids 1.1 [74], using the same settings as in [29].

### Statistical analyses

#### Parasite: spatio-temporal variation

We applied a principal component analysis (PCA) on the entire parasite sequence dataset (i.e., 16 population samples that were genotyped after pooling of infected *Daphnia,* and 3 of these population samples in which infected *Daphnia* were also genotyped individually, see Table 1). The PCA was calculated in SPSS 20.0 (using varimax rotation) and was based on the frequencies of *Caullerya* representative ITS-sequence variants (TCS-types) in each sample. Then, to test for statistical differentiation, we applied hierarchical analyses of molecular variance, AMOVA (distance method and pairwise differences, calculated in Arlequin [75]), on subsets of parasite data. Thus, to test for spatial patterns, we partitioned the *Caullerya* genetic variation into two components: 1) among lakes, and 2) within lake. AMOVA was applied separately per year, focusing on years with more than two spatially isolated population samples with sufficient *Caullerya* prevalence (i.e., 2004, 2005 and 2009; Table 1). Similarly, to test for temporal patterns, genetic variation was partitioned into two components: 1) among years, and 2) within year. Here, AMOVA was applied separately per lake, focusing on lakes with more than two temporarily isolated population samples with sufficient *Caullerya* prevalence (i.e., Římov, Vír and Vranov; Table 1). The significance of each AMOVA run was assessed relative to 1000 randomly permuted datasets. Sequential Bonferroni corrections [76] were applied to adjust significance levels from separate analyses concerning space and time, respectively.

#### Parasite and host: genetic associations

Here, we used ITS parasite data combined with microsatellite data from hosts, obtained from 30 host individuals sampled in 2004 from Římov, Vír, and Želivka (3 lakes × 10 individuals, Table 1). Host taxon identity was assigned based on the position of individuals in the FCA in relation to reference genotypes (see Additional file 1: Figure S1). First, we compared (by Fisher’s exact tests in SPSS 20.0) the distribution of parasite representative sequence variants (TCS types): (a) among (abundant) host species within lakes, and (b) among lakes within each single (abundant) host species. Second, to determine the association between host species and certain parasite genotypes, we applied AMOVA, partitioning the parasite genetic variation into three components: 1) among lakes, 2) among host species within-lakes, and 3) within host species.

#### Host: spatial variation

Based on the microsatellite screening of ~50 randomly selected *Daphnia* individuals from Římov, Vír and Želivka sampled in 2004, the only host species present in all three localities was *D. galeata*. Thus, an AMOVA test partitioning the host genetic variance into two components (i.e., among lakes and within lake) was performed for this species only.

## Availability of supporting data

The ITS data set supporting the results of this article has been published elsewhere [40] and is available in the DRYAD repository: doi:10.5061/dryad.8c1d0 (DNA alignment of 795 ITS sequences) and in the GenBank: accession no HQ219692–HQ219708 (ITS types). The microsatellite data have been deposited in DRYAD: doi:10.5061/dryad.6773h.

## Additional file

Additional file 1: Figure S1. Factorial correspondence analysis (FCA) showing taxon assignment and genetic similarity among 30 infected *Daphnia* host individuals originating from three lakes sampled in 2004 (Římov, Vír and Želivka; see Table 1). Additionally, 49 reference clones are shown (black dots, for a list of reference clones see [43]). Loadings on the FCA-axes are based on the frequency of allelic variation at 15 microsatellite loci; the first two axes account for 16% of the variation in the data: cuc – *D. cucullata*, gal – *D. galeata*, lon – *D. longispina* (their respective hybrids are also shown).

## References

[1] Koskella B, Lively CM (2007). Advice of the rose: experimental coevolution of a trematode parasite and its snail host. Evolution.

[2] Schulte RD, Makus C, Hasert B, Michiels NK, Schulenburg H (2011). Host-parasite local adaptation after experimental coevolution of *Caenorhabditis elegans* and its microparasite *Bacillus thuringiensis*. Proc R Soc B.

[3] Morran LT, Schmidt OG, Gelarden IA, Parrish RC, Lively CM (2011). Running with the Red Queen: host-parasite coevolution selects for biparental sex. Science.

[4] Wolinska J, King KC (2009). Environment can alter selection in host-parasite interactions. Trends Parasitol.

[5] Mostowy R, Engelstadter J (2011). The impact of environmental change on host-parasite coevolutionary dynamics. Proc R Soc B.

[6] Little TJ, Ebert D (1999). Associations between parasitism and host genotype in natural populations of *Daphnia* (Crustacea: Cladocera). J Anim Ecol.

[7] Wolinska J, Spaak P (2009). The cost of being common: evidence from natural *Daphnia* populations. Evolution.

[8] Decaestecker E, Gaba S, Raeymaekers JAM, Stoks R, Van Kerckhoven L, Ebert D, De Meester L (2007). Host-parasite "Red Queen" dynamics archived in pond sediment. Nature.

[9] Gsell AS, de Senerpont Domis LN, Verhoeven KJF, Van Donk E, Ibelings BW (2013). Chytrid epidemics may increase genetic diversity of a diatom spring-bloom. ISME J.

[10] Vernon JG, Okamura B, Jones CS, Noble LR (1996). Temporal patterns of clonality and parasitism in a population of freshwater bryozoans. Proc R Soc B.

[11] Siemens DH, Roy BA (2005). Tests for parasite-mediated frequency-dependent selection in natural populations of an asexual plant species. Evol Ecol.

[12] Burdon JJ, Thompson JN (1995). Changed patterns of resistance in a population of *Linum marginale* attacked by the rust pathogen *Melampsora lini*. J Ecol.

[13] Jokela J, Dybdahl ME, Lively CM (2009). The maintenance of sex, clonal dynamics, and host-parasite coevolution in a mixed population of sexual and asexual snails. Am Nat.

[14] King KC, Delph LF, Jokela J, Lively CM (2009). The geographic mosaic of sex and the Red Queen. Curr Biol.

[15] Jaenike J (1978). A hypothesis to account for the maintenance of sex within populations. Evol Theory.

[16] Hamilton WD (1980). Sex versus non-sex versus parasite. Oikos.

[17] Sarkar SF, Guttman DS (2004). Evolution of the core genome of *Pseudomonas syringae*, a highly clonal, endemic plant pathogen. Appl Environ Microbiol.

[18] Orjuela-Sanchez P, Da Silva-Nunes M, Da Silva NS, Scopel KKG, Goncalves RM, Malafronte RS, Ferreira MU (2009). Population dynamics of genetically diverse *Plasmodium falciparum* lineages: community-based prospective study in rural Amazonia. Parasitology.

[19] Ghedin E, Sengamalay NA, Shumway M, Zaborsky J, Feldblyum T, Subbu V, Spiro DJ, Sitz J, Koo H, Bolotov P, Dernovoy D, Tatusova T, Bao Y, St George K, Taylor J, Lipman DJ, Fraser CM, Taubenberger JK, Salzberg SL (2005). Large-scale sequencing of human influenza reveals the dynamic nature of viral genome evolution. Nature.

[20] Schall JJ, Denis KMS (2013). Microsatellite loci over a thirty-three year period for a malaria parasite (*Plasmodium mexicanum*): bottleneck in effective population size and effect on allele frequencies. Parasitology.

[21] Thrall PH, Laine A-L, Ravensdale M, Nemri A, Dodds PN, Barrett LG, Burdon JJ (2012). Rapid genetic change underpins antagonistic coevolution in a natural host-pathogen metapopulation. Ecol Lett.

[22] Flor HH (1956). The complementary genetic systems in flax and flax rust. Adv Genet.

[23] Dodds PN, Rathjen JP (2010). Plant immunity: towards an integrated view of plant-pathogen interactions. Nat Rev Genet.

[24] Gandon S, Capowiez Y, Dubois Y, Michalakis Y, Olivieri I (1996). Local adaptation and gene-for-gene coevolution in a metapopulation model. Proc R Soc B.

[25] Gandon S (2002). Local adaptation and the geometry of host-parasite coevolution. Ecol Lett.

[26] Lively CM (1996). Host-parasite coevolution and sex: do interactions between biological enemies maintain genetic variation and cross-fertilization?. Bioscience.

[27] Duffy MA, Ochs JH, Penczykowski RM, Civitello DJ, Klausmeier CA, Hall SR (2012). Ecological context influences epidemic size and parasite-driven evolution. Science.

[28] Duncan A, Little TJ (2007). Parasite-driven genetic change in a natural population of *Daphnia*. Evolution.

[29] Yin M, Petrusek A, Seda J, Wolinska J (2012). Fine-scale genetic analysis of *Daphnia* host populations infected by two virulent parasites – strong fluctuations in clonal structure at small temporal and spatial scales. Int J Parasitol.

[30] Schwarzenberger A, D'Hondt S, Vyverman W, von Elert E (2013). Seasonal succession of cyanobacterial protease inhibitors and *Daphnia magna* genotypes in a eutrophic Swedish lake. Aquat Sci.

[31] Haag KL, Ebert D (2012). Single-nucleotide polymorphisms of two closely related microsporidian parasites suggest a clonal population expansion after the last glaciation. Mol Ecol.

[32] Haag KL, Sheikh-Jabbari E, Ben-Ami F, Ebert D (2013). Microsatellite and single-nucleotide polymorphisms indicate recurrent transitions to asexuality in a microsporidian parasite. J Evol Biol.

[33] Schwenk K, Spaak P (1995). Evolutionary and ecological consequences of interspecific hybridization in cladocerans. Experientia.

[34] Petrusek A, Hobaek A, Nilssen JP, Skage M, Cerny M, Brede N, Schwenk K (2008). A taxonomic reappraisal of the European *Daphnia longispina* complex (Crustacea, Cladocera, Anomopoda). Zool Scr.

[35] Wolinska J, Seda J, Koerner H, Smilauer P, Petrusek A (2011). Spatial variation of *Daphnia* parasite load within individual water bodies. J Plankton Res.

[36] Lohr J, Laforsch C, Koerner H, Wolinska J (2010). A *Daphnia* parasite (*Caullerya mesnili*) constitutes a new member of the Ichthyosporea, a group of protists near the animal-fungi divergence. J Eukaryot Microbiol.

[37] Wolinska J, Bittner K, Ebert D, Spaak P (2006). The coexistence of hybrid and parental *Daphnia*: the role of parasites. Proc R Soc B.

[38] Wolinska J, Spaak P, Petrusek A, Koerner H, Seda J, Giessler S (2011). Transmission mode affects the population genetic structure of *Daphnia* parasites. J Evol Biol.

[39] Bittner K, Rothhaupt KO, Ebert D (2002). Ecological interactions of the microparasite *Caullerya mesnili* and its host *Daphnia galeata*. Limnol Oceanogr.

[40] Giessler S, Wolinska J (2013). Capturing the population structure of microparasites: using ITS-sequence data and a pooled DNA approach. Mol Ecol Res.

[41] Ventura M, Petrusek A, Miro A, Hamrová E, Bunay D, De Meester L, Mergeay J (2014). Local and regional founder effects in lake zooplankton persist after thousands of years despite high dispersal potential. Mol Ecol.

[42] Hamrová E, Mergeay J, Petrusek A (2011). Strong differences in the clonal variation of two Daphnia species from mountain lakes affected by overwintering strategy. BMC Evol Biol.

[43] Yin M, Wolinska J, Giessler S (2010). Clonal diversity, clonal persistence and rapid taxon replacement in natural populations of species and hybrids of the *Daphnia longispina* complex. Mol Ecol.

[44] Havel JE, Shurin JB (2004). Mechanisms, effects, and scales of dispersal in freshwater zooplankton. Limnol Oceanogr.

[45] Figuerola J, Green AJ, Michot TC (2005). Invertebrate eggs can fly: evidence of waterfowl-mediated gene flow in aquatic invertebrates. Am Nat.

[46] Louette G, Vanoverbeke J, Ortells R, De Meester L (2007). The founding mothers: the genetic structure of newly established *Daphnia* populations. Oikos.

[47] Ortells R, Vanoverbeke J, Louette G, De Meester L (2014). Colonization of Daphnia magna in a newly created pond: founder effects and secondary immigrants. Hydrobiologia.

[48] Ebert D, Zschokke Rohringer CD, Carius HJ (2000). Dose effects and density-dependent regulation of two microparasites of *Daphnia magna*. Oecologia.

[49] Mendoza L, Taylor JW, Ajello L (2002). The class mesomycetozoea: a group of microorganisms at the animal-fungal boundary. Annu Rev Microbiol.

[50] Marshall WL, Berbee ML (2010). Population-level analyses indirectly reveal cryptic sex and life history traits of *Pseudoperkinsus tapetis* (Ichthyosporea, Opisthokonta): a unicellular relative of the animals. Mol Biol Evol.

[51] Schoebel CN, Tellenbach C, Spaak P, Wolinska J (2011). Temperature effects on parasite prevalence in a natural hybrid complex. Biol Lett.

[52] Agrawal A, Lively CM (2002). Infection genetics: gene-for-gene versus matching-alleles models and all points in between. Evol Ecol Res.

[53] Lythgoe KA (2002). Effects of acquired immunity and mating strategy on the genetic structure of parasite populations. Am Nat.

[54] Schmid-Hempel P, Funk CR (2004). The distribution of genotypes of the trypanosome parasite, *Crithidia bombi*, in populations of its host, *Bombus terrestris*. Parasitology.

[55] Erler S, Popp M, Wolf S, Lattorff HMG (2012). Sex, horizontal transmission, and multiple hosts prevent local adaptation of *Crithidia bombi*, a parasite of bumblebees (*Bombus* spp.). Ecol Evol.

[56] Salathe RM, Schmid-Hempel P (2011). The genotypic structure of a multi-host bumblebee parasite suggests a role for ecological niche overlap. Plos One.

[57] Seda J, Petrusek A, Machacek J, Smilauer P (2007). Spatial distribution of the *Daphnia longispina* species complex and other planktonic crustaceans in the heterogeneous environment of canyon-shaped reservoirs. J Plankton Res.

[58] Petrusek A, Seda J, Machacek J, Ruthova S, Smilauer P (2008). *Daphnia* hybridization along ecological gradients in pelagic environments: the potential for the presence of hybrid zones in plankton. Philos Trans R Soc Lond B.

[59] Floate KD, Whitham TG (1993). The hybrid bridge hypothesis: host shifting via plant hybrid swarms. Am Nat.

[60] Holt RD, Dobson AP, Begon M, Bowers RG, Schauber EM (2003). Parasite establishment in host communities. Ecol Lett.

[61] Dobson A (2004). Population dynamics of pathogens with multiple host species. Am Nat.

[62] Archie EA, Ezenwa VO (2011). Population genetic structure and history of a generalist parasite infecting multiple sympatric host species. Int J Parasitol.

[63] Andras JP, Ebert D (2013). A novel approach to parasite population genetics: experimental infection reveals geographic differentiation, recombination and host-mediated population structure in *pasteuria ramosa*, a bacterial parasite of *daphnia*. Mol Ecol.

[64] Carriconde F, Gardes M, Jargeat P, Heilmann-Clausen J, Mouhamadou B, Gryta H (2008). Population evidence of cryptic species and geographical structure in the cosmopolitan ectomycorrhizal fungus, *Tricholoma Scalpturatum*. Microb Ecol.

[65] Stukenbrock EH, McDonald BA (2009). Population genetics of fungal and oomycete effectors involved in gene-for-gene interactions. Mol Plant-Microbe Interact.

[66] Stahl EA, Dwyer G, Mauricio R, Kreitman M, Bergelson J (1999). Dynamics of disease resistance polymorphism at the *Rpm1* locus of *Arabidopsis*. Nature.

[67] Tollenaere C, Susi H, Nokso-Koivisto J, Koskinen P, Tack A, Auvinen P, Paulin L, Frilander MJ, Lehtonen R, Laine A-L (2012). SNP design from 454 sequencing of *Podosphaera plantaginis* transcriptome reveals a genetically diverse pathogen metapopulation with high levels of mixed-genotype infection. Plos One.

[68] Qi W, Kaeser M, Roeltgen K, Yeboah-Manu D, Pluschke G (2009). Genomic diversity and evolution of *Mycobacterium ulcerans* revealed by next-generation sequencing. PLoS Pathog.

[69] Schoebel CN, Jung E, Prospero S (2013). Development of new polymorphic microsatellite markers for three closely related plant-pathogenic *Phytophthora* species using 454-pyrosequencing and their potential applications. Phytopathology.

[70] Hall TA (1999). BioEdit: a user-friendly biological sequence alignment editor and analysis program for Windows 95/98/NT. Nucleic Acids Symp Ser.

[71] Clement M, Posada D, Crandall KA (2000). TCS: a computer program to estimate gene genealogies. Mol Ecol.

[72] Brede N, Thielsch A, Sandrock C, Spaak P, Keller B, Streit B, Schwenk K (2006). Microsatellite markers for European *Daphnia*. Mol Ecol Notes.

[73] Bélkhir K, Borsa P, Chikhi L, Raufaste N, Bonhomme F: **GENETIX 4.05, logiciel sous windows TM pour la génétique des populations. Laboratoire génome, populations, interactions, CNRS UMR 5000, université de Montpellier II, Montpellier (France).**Available from URL: http://kimura.univ-montp2.fr/genetix/.

[74] Anderson EC, Thompson EA (2002). A model-based method for identifying species hybrids using multilocus genetic data. Genetics.

[75] Excoffier L, Lischer HEL (2010). Arlequin suite ver 3.5: a new series of programs to perform population genetics analyses under Linux and windows. Mol Ecol Res.

[76] Rice WR (1989). Analyzing tables of statistical tests. Evolution.

