# Supplementary figures and images for: Population structure of a microparasite infecting Daphnia: spatio-temporal dynamics
Source: BMC Evol Biol. 2014 Dec 4;14:247. doi: 10.1186/s12862-014-0247-3 (PMC4265321; doi:10.1186/s12862-014-0247-3)

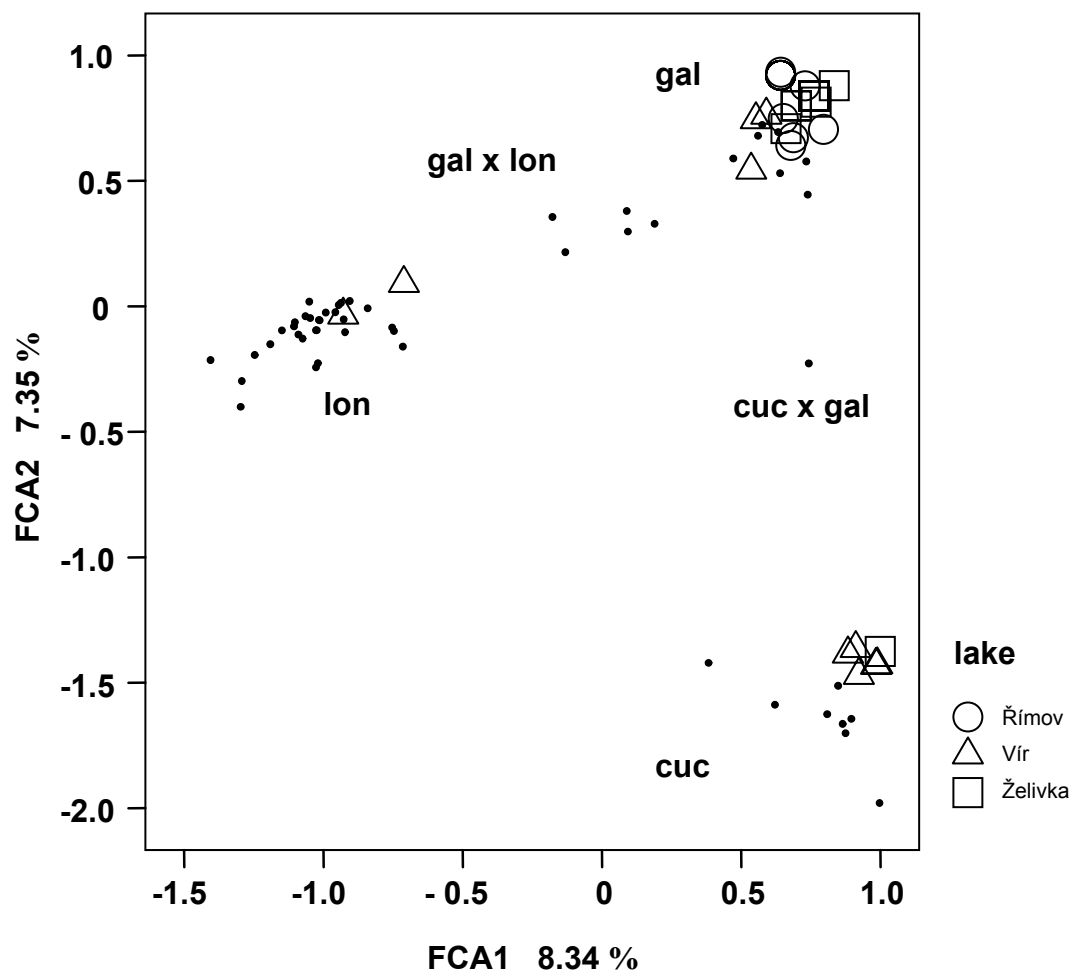

Supplement: Additional file 1: Figure S1. — Factorial correspondence analysis (FCA) showing taxon assignment and genetic similarity among 30 infected Daphnia host individuals originating from three lakes sampled in 2004 (Římov, Vír and Želivka; see Table 1). Additionally, 49 reference clones are shown (black dots, for a list of reference clones see [43]). Loadings on the FCA-axes are based on the frequency of allelic variation at 15 microsatellite loci; the first two axes account for 16% of the variation in the data: cuc – D. cucullata, gal – D. galeata, lon – D. longispina (their respective hybrids are also shown). [file 12862_2014_247_MOESM1_ESM.pdf]
